# A multi-center, randomized controlled clinical trial of the application of a shortened protocol of long-acting Triptorelin down-regulated prior to IVF/ICSI among patients with endometriosis: A protocol

**DOI:** 10.1186/s12978-018-0639-8

**Published:** 2018-12-20

**Authors:** Huijuan Kong, Linli Hu, Ling Nie, Xiaona Yu, Wei Dai, Jing Li, Caihong Chen, Zhiqin Bu, Hao Shi, Qiongfang Wu, Yichun Guan, Yingpu Sun

**Affiliations:** 1 Center for Reproductive Medicine, the First Affiliated Hospital of Zhengzhou University, Zhengzhou, China; 2 Henan Key Laboratory of Reproduction and Genetics, the First Affiliated Hospital of Zhengzhou University, Zhengzhou, China; 3 Jiangxi Maternal and Child Health hospital, Nanchang, China; 4 Center for Reproductive Medicine, the Third Affiliated Hospital of Zhengzhou University, Zhengzhou, China

## Abstract

**Background:**

Endometriosis is the major cause of progressive pelvic pain and subfertility. Up to 50% of reproductive-age women suffer from pelvic pain. Endometriosis is a classic indication for IVF. Compared with women whose inability to procreate is caused by simple tubal infertility, women with endometriosis often have lower pregnancy rates following in vitro fertilization/intracytoplasmic sperm injection (IVF/ICSI). The administration of gonadotrophin-releasing hormone (GnRH) agonists prior to IVF/ICSI can improve the successful pregnancy rate. Whether a briefer treatment interval would be efficacious has not been studied.

**Methods/design:**

Eligible and consenting women will be randomly assigned to one of two treatments (one cycle of a GnRH agonist or two cycles of a GnRH agonist) prior to IVF/ICSI using a table of random numbers. The primary outcome of this trial is clinical pregnancy rate. Other outcomes include gonadotrophin (Gn) duration, the total dose of follicle-stimulating hormone (FSH) used, number of oocytes retrieved, number of embryos available for transfer, implantation rate, the abortion rate, live birth rate, and incidence of moderate-to-severe ovarian hyperstimulation. The sample size of this trial is estimated to be 421 participants for each of the two arms. Appropriate interim analyses will be conducted by a data monitoring and ethics committee (DMEC), and the final test will be an intention-to-treat analysis.

**Trial registration:**

This trial has been assigned the following registry number: NCT03006406.

## Plain English summary

Endometriosis is a disease causing progressive pelvic pain and infertility. Up to 50% of reproductive-age women suffer from dysmenorrhea, sexual pain. Surgery or hormone therapy may be meaningful for pain, however, ovary function may be damaged after laparascopy. Progestin and androgen therapy or the oral contraceptive were unhelpful for pragnancy while unexpectedly creating more side effects.

Endometriosis is a classic indication for in vitro fertilization (IVF). Compared with women whose inability to procreate is caused by simple tubal infertility, women with endometriosis often have lower pregnancy rates. The administration of long acting gonadotrophin-releasing hormone (GnRH) agonists prior to IVF/ICSI can improve the successful pregnancy rate. A typical ultra-long-acting regimen may create a low estrogen status of menopause. Such as hectic fever, night sweating and loss of bone mass are significant Whether a briefer treatment interval would be efficacious has not been studied.

Patients were invited to different treatment groups according to random number table. The rest part of IVF treatment along to routine procedure in three reproductive centers.

The top research priorities identified were: the fresh embryo transfer rate, pregnancy rate, total Gn and other indicators; more effective treatments; outcomes and severely affected patients. Least popular priorities were: economic research towards the cost of IVF; and psychological aspects.

In conclusion; individuals affected by endometriosis will have a new view that they could suffer less discomfort and get a live birth quickly. We aimed to provide a safer and more effective ovulation induction regimen for patients with endometriosis.

## Background

Endometriosis is defined as the presence of endometrial-like tissue outside the uterine cavity. This chronic inflammatory condition is estrogen dependent. The incidence and prevalence rates are difficult to quantify because they depend on surgical visualization for a precise diagnosis. Wide ranges have been reported in the literature; 6–10% of reproductive-age woman suffer from endometriosis, women with endometriosis have dysmenorrhea, and up to 50% of women are infertile [9]. Women with pelvic pain identified via endometriosis laparoscopy were followed up; 24–64% of lesions were progressive, and 9–59% were stable over 12 months [15]. The incidence of endometriosis peaks between 25 and 35 years old, which is the greatest period of reproduction [17].

Infertility is common among women with endometriosis. Most reasons are related to chronic pelvic inflammation [5]. Pelvic adhesions can disrupt the pelvic anatomy and alter peritoneal function, fallopian tubal transport is often abnormal, and inflammatory molecules can form under abnormal circumstances that are unfavorable for pregnancy. A tangled anatomy can be repaired via surgery to repair pelvic adhesions and tubal non-function. IVF is a good choice for attempting pregnancy.

Compared with women with tubal infertility, women with endometriosis have lower IVF/ICSI success rates. One meta-analysis that included 22 non-randomized controls reported that the infertility of women with endometriosis was lower than that of those with tubal infertility (odds ratio [OR] = 0.56, 95% confidence intervals [CIs] = 0.44–0.7; [1]). A multivariate analysis also showed that the fertilization and implantation rates of women with endometriosis were significantly lower than those of women receiving the same number of eggs. The low rates of pregnancy and implantation are primarily due to poor egg quality. Poor egg quality leads to a low fertilization rate, which in turn affects the quality of the embryo and affects the implantation rate, especially in patients with severe endometriosis. In oocyte donation cycles, when healthy women donate their disease-free eggs to women with endometriosis, the recipient is associated with comparative endometrial receptivity and pregnancy rates. When the oocytes of women with endometriosis are donated to disease-free women, their implantation rates are lower [6]. Severe endometriosis is associated with a lower pregnancy rate than mild endometriosis (OR = 0.60, 95% CIs = 0.42–0.87; [1]). A meta-analysis provided statistical evidence of the above statement, finding relative pregnancy rates of 0.93 (95% CIs = 0.87–0.99) and 0.79 (96% CIs = 0.69–0.91) among women with mild and severe endometriosis, respectively, through IVF [10].

No clear mechanism has been defined to explain the relationship between endometriosis and subfertility. The complex mechanisms of ovulation, fertilization, tubal pick-up oocyte, and the transport of the embryos or embryo implantation might be partially influenced by hormones [5]. Elevated cytokine and progesterone concentrations in the follicular fluid were found in women with endometriosis [7]. This theory is supported by findings showing concentrations of prostaglandins, proteases, and inflammatory cytokines in the peritoneal fluid of women with endometriosis [2].

As such, reproductive experts have sought to clarify the best IVF treatment protocol for women with endometriosis. The administration of GnRH agonists prior to IVF/ICSI improves the pregnancy rate. The 2006 edition of the Cochrane guidelines recommend the use of 2–6 months of GnRH analogs before the initiation of an IVF cycle to “turn off the inflammatory response”, thereby increasing the pregnancy rate. One meta-analysis that included the randomized controlled trial (RCT) results comparing a general regimen with an ultra-long-acting regimen across three small-scale samples showed that the OR of the pregnancy rate was 4.28 (95% CIs = 2.00–9.15; [13]). Benschop did not find a difference in the pregnancy rates between GnRH agonists and GnRH antagonists administered prior to ART (OR = 0.81, 95% CIs = 0.26–2.54; 67 participants, one trial; [3]). Three additional reviews observed similar benefits for the oral administration of contraceptives versus no treatment [11]; these results require more corroborating evidence. In all cases, the quality of these trials was generally poor, and adequate allocation was concealed. These trials were also generally small, leading to imprecise findings, even when combined in a meta-analysis [4]. The conclusion of the most recent research was that long-term pituitary down-regulation has only a limited effect after including cryopreserved embryo transfers among women with severe endometriosis [16].

A typical ultra-long-acting regimen is to administer 2–3 doses of long-acting Triptorelin prior to ovulation induction to create a low estrogen status of menopause, which allows the ectopic endometrium to atrophy, and ovulation induction is then carried out by gonadotropin (Gn). However, although the patients with endometriosis who use this regimen for ovulation induction can achieve a certain pregnancy rate, the treatment cycle is long, the side effects of low estrogen (such as hectic fever, night sweating and loss of bone mass) are significant, and the use of gonadotropins is high. Therefore, how to ensure a good pregnancy rate while reducing the side effects during the treatment, the treatment cycle, the use of the treatment drug, and the treatment cost has become a focus of ovulation induction research for patients with endometriosis. Whether a briefer treatment interval is efficacious has not been studied [14]. No trials have compared a shorter interval with the typically long protocol.

In this study, we will use an RCT to compare the fresh embryo transfer rate, pregnancy rate, total Gn and other indicators during the ovulation induction application of the ultra-long-acting regimen with one dose or two doses of long-acting Triptorelin in patients with endometriosis, aiming to provide a safer and more effective ovulation induction regimen for patients with endometriosis.

Centers involved in the study:

1. Center for Reproductive Medicine, the First Affiliated Hospital of Zhengzhou University, Zhengzhou, China

2. Jiangxi Provincial Maternal and Child Health Hospital in Nanchang

3. Center for Reproductive Medicine, Third Affiliated Hospital of Zhengzhou University, Zhengzhou, China

## Methods/design

### Aim

In this study, we hypothesis that that the efficacy of using one dose of long-acting GnRH agonist is no worse than that of two doses of GnRH agonist when carrying out IVF-assisted pregnancy for patients with combined endometriosis. At the same time, the treatment cycle is shortened and the total dose of Gn used is reduced.

### Design

A prospective, multicenter, single-blinded, randomized controlled study.

### Main research objective

Clinical pregnancy rate is no worse than that of a control group.

### Secondary research objectives

1. The treatment cycle is shortened.

2. The amount of Gn is reduced.

### Research endpoints

#### Main study endpoint

1. Clinical pregnancy rate (%)

#### Secondary study endpoints

1. Estradiol (E2) level on the day of human chorionic gonadotrophin (HCG) administration

2. Progesterone (P) level on the day of HCG administration

3. The number of oocytes retrieved

4. Gn dosage

5. Gn duration

6. Metaphase II (MII) ratio (during ICSI injection) (%)

7. Two-pronuclei (2PN) ratio (%)

8. Embryos available for transfer

9. Ratio of fresh cycle to transfer cycle (%)

10. Incidence of moderate-to-severe ovarian hyperstimulation syndrome (OHSS) (%)

11. Implantation rate (%)

12. Early miscarriage rate

13. Ectopic pregnancy rate

14. Ongoing pregnancy rate

15. Live birth rate

16. Multiple pregnancy rate

### Inclusion/exclusion criteria

All subjects must meet all inclusion criteria and must not meet any of the exclusion criteria.

#### Inclusion criteria

1. Infertile women younger than 40 years old;

2. Pelvic endometriosis lesions confirmed by laparoscopy or laparotomy, which is described in detail in the surgical records and can be scored;

3. Meet the indications to receive IVF/ICSI treatment;

4. Normal blood FSH levels (FSH < 12 IU/L);

5. In line with the national family planning policy;

6. The patient signed the informed consent and was willing to complete the follow-up on time.

#### Exclusion criteria

1. Patient with alcoholism, tobacco addiction, or drug abuse habits;

2. Patient with any serious cardiovascular, lung, liver, or kidney diseases;

3. Patient with contraindications to pregnancy;

4. Patient with preimplantation genetic diagnosis (PGD) or preimplantation genetic screening (PGS);

5. Patient with other ovarian surgical history other than endometriosis surgery;

6. Patients with uterine fibroids (fibroids with a diameter larger than 3 cm or closely related to the endometrium);

7. Patients with hydrosalpinx;

8. Patients with adenomyosis;

9. Patients with untreated endometrial polyps;

10. Patients with intrauterine adhesions;

11. Pap smear test (ThinPrep cytologic test, TCT) indicating stage III/IV within 6 months;

12. Number of previous IVF/ICSI attempts ≥3;

13. Patients participating in any other clinical trials.

### Grouping method

Center controlled grouping will be performed. After entering the IVF/ICSI cycle and establishing medical records, the researchers at each center will screen for patients who meet the enrollment requirements according to the inclusion and exclusion criteria. Patients with endometriosis will be scored, and the degree of endometriosis (mild, moderate or severe) will be evaluated and recorded in a Microsoft Excel spreadsheet for each center. At the same time, the researchers at the center will be contacted, and the statisticians at the center will group the patients according to the number randomly generated by Excel or contact the central office.

### Blind method

The study is single-blinded; the doctor who prescribes the drug for ovulation induction will determine the ovulation induction regimen. Both physicians for Ovum pick up and embryo transfer, and embryologist handling embryos are blinded in this study.

## Method

**Fig. 1:**
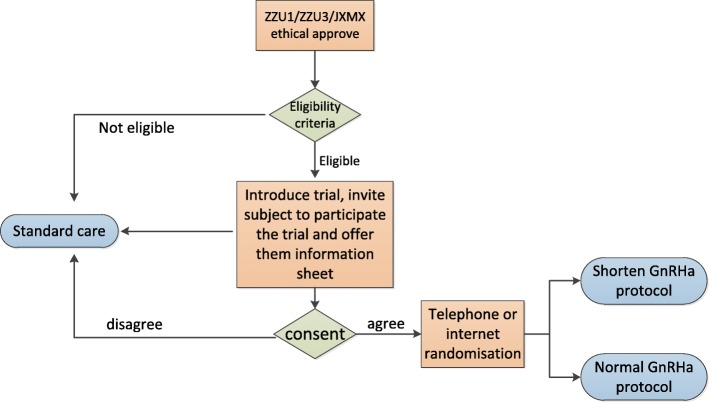
Participant flow chart through the trial

All women who are confirmed to have endometriosis by laparotomy or laparoscopic surgery will visit the reproductive center and receive assisted pregnancy treatment. After the patients enter the IVF/ICSI cycle and establish medical records, the researchers at each center will screen patients who meet the enrollment requirements according to the inclusion and exclusion criteria. The patients will be fully informed about why they were enrolled in the study. If the patients are highly satisfied with the explanation about this research and decide to participate in the study after careful consideration, they will be asked to sign an informed consent form. Patients will understand they could withdraw voluntarily from this clinical study at any time, and the person in charge at each center will provide the patient with their contact phone number. If the patient decides to withdraw, it will be recorded as lost. See Fig. 1.

### Research plan

#### The classic ultra-long-acting regimen—ultra-long-acting regimen with two injections

**Fig. 2:**
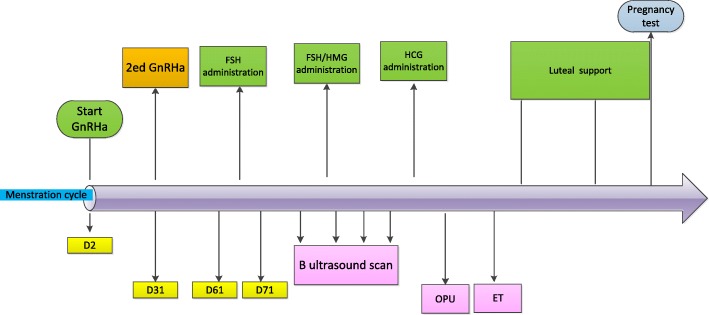
Normal GnRHa protocol

The patients will visit the hospital on menstrual day 2. If the vaginal B-scan ultrasonography does not find cysts and follicles > 10 mm, then 3.75 mg of long-acting GnRHa (Diphereline is the common choice) will be injected. The patient will attend a checkup 28 days after the injection to examine the B-scan ultrasonography+FSH, luteinizing hormone (LH), E2, and P. If ovarian endometriosis is found, then a B-scan ultrasound-guided aspiration biopsy of the cyst will be performed. An intraoperative aspiration of the cyst fluid will be obtained and saline will be used to repeatedly rinse the cyst cavity. At the same time, the second 3.75 mg of long-acting GnRHa will be injected. Twenty-eight to 30 days after the injection (to be adjusted slightly according to the diagnosis and treatment habit of the center or decided according to the patient’s condition), the patient will attend a checkup to examine the B-scan ultrasonography+FSH, LH, E2, and P to see whether they have achieved down-regulation criteria. The down-regulation criteria are E2 < 50 pg/ml, *P* < 0.8 ng/ml, LH < 3 mIU/ml, and endometrium thickness ≤ 5 mm. The researchers then determine the Gn initiation time according to follicle and hormone conditions. See Fig. 2.

#### Early follicular phase long-acting regimen—Ultra-long-acting regimen with one injection

**Fig. 3:**
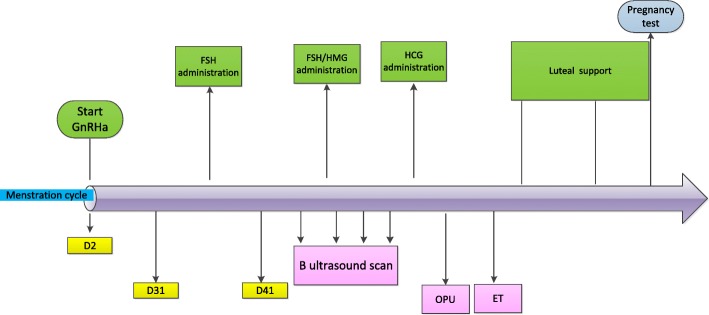
Shorten GnRHa protocol

The patient will visit the hospital on menstrual day 2. If the vaginal B-scan ultrasonography does not find cysts and follicles > 10 mm, then 3.75 mg of long-acting GnRHa (Diphereline is the common choice) will be injected. The patient will attend a checkup 28 days after the injection. If ovarian endometriosis is found, then a B-scan ultrasound-guided aspiration biopsy of the cyst will be performed. An intraoperative aspiration of the cyst fluid will be obtained, and saline will be used to repeatedly rinse the cyst cavity. On the same day, B-scan ultrasonography+FSH, LH, E2, and P will be examined. Thirty to 40 days after the injection, patients are expected to reach down-regulation criteria. The down-regulation criteria are E2 < 50 pg/ml, *P* < 0.8 ng/ml, LH < 3 mIU/ml, and endometrium thickness ≤ 5 mm. Then, Gn will be started. See Fig. 3.

#### Gn starting dose

According to the subject’s age, BMI, antral follicle number, and medical history, the researchers at each center will give a recommendation on a random date, while the details will be decided after a reevaluation by the researchers on the start date, usually 112.5–150 IU. During ovulation induction, the Gn will be increased or decreased timely according to the number, size, and growth of follicles.

### Gn adjustment

The researchers will determine the increase and decrease in Gn based on the hormone status and follicular development, and human menopausal gonadotropin (HMG) will be added in a timely manner (Menotrophin will be used in all cases).

### Criteria for HCG injection

The criteria for HCG injection include the following: when the diameter of one primary follicle is ≥20 mm and the diameter of the other follicles is ≥18 mm, or the quantity of follicles with a diameter ≥ 14 mm accounts for more than 2/3 of the follicles. The trigger drug and HCG exposure time will be determined by the researchers according to the patient’s body weight, estrogen levels, and follicle condition. Ovidrel + HCG 2000 IU trigger will be given routinely.

### Egg retrieval

Eggs will be retrieved 36–37 h after HCG administration according to the routine procedure of each center.

### Embryo transfer

On the third and fifth days after the eggs are taken, 1–2 embryos or blastocysts will be transferred depending on the quantity and quality of the embryos.

### Luteal support

All women undergoing IVF-assisted pregnancy will start luteal support once the eggs are taken. They will be treated with Crinone (Switzerland, Merck-Serono) 400 mg/day, and Duphaston tablets (USA, Abbott) 20 mg/day until the early pregnancy test. If the test is positive, the administration of both drugs will be continued. Crinone will be stopped at 45 days after the transfer, while Duphaston will be stopped at 65 days after the transfer.

### Pregnancy test

Blood and urine HCG will be routinely measured 14 days after embryo transfer.

### Ultrasound test of early pregnancy

Thirty-five days after the embryo transfer, abdominal ultrasound will be used to determine the size of the gestational sac, fetal bud, and the primitive heart tube beat.

### Criteria for canceling embryo transfer

1. *P* > 3 ng/ml on the day of HCG administration;

2. Presence of uterine effusion on the day of transfer;

3. Moderate to severe ovarian hyperstimulation criteria are met on the day of transfer according to the OHSS grading criteria [8]:

### Study steps and observation parameters

#### Study steps

##### Visit 1

Screen for subjects based on inclusion/exclusion criteria;

Explain the study to the subjects and have the subjects sign the informed consent form;

Collect medical history and demographic information and perform routine physical examination (including height and weight) and pelvic examination;

Count antral follicles;

Baseline anti-Müllerian hormone (AMH), FSH, LH, E2, testosterone (T), P, and prolactin (PRL) levels in serum.

##### Visit 2 (down-regulation and ovarian stimulation)

Record the time to start the down-regulation treatment;

Record the serum FSH, LH, E2, and P levels after down-regulation but before giving Gn;

Record the number and size of antral follicles after down-regulation but before administration Gn;

Initiation dose: 112.5–150 IU, maximum dose 300 IU.

##### Visit 3 (Gn 6–7 days)

Record the serum LH, E2, and P levels for 7 days;

Record the size and number of follicles.

##### Visit 4 (the day HCG was administered)

HCG medication regimen and time: Once there is evidence to show that follicular development was mature; Ovidrel + HCG 2000 IU trigger will be given. If the patient has OHSS tendencies, we will refer to the diagnosis and treatment routine of our center to give Ovidrel or HCG (3000–4000 IU) to minimize the occurrence of serious complications.

Record the total amount of Gn and medication time;

Record the E2 level;

Record the number of follicles < 14 mm, 14–16 mm and > 16 mm;

If the cycle is canceled (if no HCG was given), the reason for stopping the treatment should be recorded in the case report form, and this visit will become the last visit for that subject.

##### Visit 5 (embryo transfer)

According to the assisted reproduction treatment plan of our center, egg retrieval, IVF/ICSI, embryo transfer, and luteal phase support will be performed.

The following data will be collected:

Gn category;

Gn dosage;

E2 and P levels;

The numbers of follicles ≥14 mm, ≥16 mm, and ≥ 18 mm;

The date the eggs were taken;

The number of eggs taken;

Oocyte insemination date;

ART method: IVF or ICSI;

Number of fertilized oocytes;

Number of 2PN eggs;

2PN cleavage rate;

Good-embryo rate;

The date of embryo transfer;

The number of transferred embryos;

The number of cryopreserved embryos;

The size of ovary;

Pelvic effusion volume;

If OHSS whole embryo freezing occurs, the OHSS level, whether the patient was hospitalized, the length of hospitalization and the outcome should be recorded;

If no embryo transfer was performed, the reason should be recorded in the case report form and this visit will become the last visit for that subject.

##### Visit 6 Biochemical pregnancy assessments

Fourteen days after embryo transfer, blood HCG will be tested. If the result is positive, then 5 weeks after the embryo transfer ultrasound examination will be conducted to evaluate the pregnancy. If the result is negative, this visit will become the last visit for that subject.

##### Visit 7 Clinical pregnancy assessments

The number of gestational sacs, the number of gestational sacs with embryonic bud and embryonic heartbeat, and the pregnancy position will be assessed by ultrasound examination. If there is no clinical gestation, then this visit will be the last visit for that subject.

##### Visit 8 Evaluation of continuous pregnancy

Approximately 12 weeks after embryo transfer, the continuous pregnancy status will be followed-up, and the presence or absence of early miscarriage will be recorded.

##### Visit 9 Evaluation of live birth rate

Approximately 40 weeks after transfer, the live birth rate will be followed up, and the presence or absence of late miscarriage, premature birth, and the health of newborns will be recorded. This visit or follow-up will be the last visit.

### End of study

The study will be considered complete when all of the following conditions are met:

The ovarian stimulation cycle was canceled (if HCG was not given);

No eggs were obtained;

No transfer;

Confirmed pregnancy status.

### Data statistics

#### Sample size of participants

This study is a non-inferiority study; that is, it works well when the clinical pregnancy rate does not significantly differ between the two arms. A statistical expert calculated the trial’s sample size based on the existing average IVF cycle outcome across the three reproductive centers and the differences in the outcomes within the existing literature. A total of 401 women in each group will achieve 90% power to detect a non-inferiority marginal difference between group proportions of − 0.0500. The control group proportion is 0.6000. The treatment group proportion is assumed to be 0.5500 under the null hypothesis of inferiority. Power was computed for cases in which the actual treatment group proportion is 0.6500. The test statistic is the one-tailed Z-test (unpooled). The significance level of the test is targeted at 0.0500. The significance level actually achieved by this design is NA [12, 18]. Considering a dropout rate of 5%, we plan to recruit 842 women in total.

#### Statistical analysis

Statistics will be treated rigorously according to the following steps:

Step 1: Summary of research data

Baseline characteristics and pregnancy results will be summarized, such as number of participants, withdrawal rate. For continuous variables (such as age, FSH level, the number of Gn days, and total Gn dose), mean and standard error will be shown, if the distribution is asymmetric, then median and quartile will be determined. For categoricalvariables (such as clinical pregnancy outcomes), percentage of different groups will be displayed.

Step 2: Comparison between groups

Chi-square test, fisher’s exact test, Mann-Whitney U testor the Kruskal-Wallis test will be usedto determine whether differences in the results of two groups have statistical significance (*p* value < 0.05). Subgroup analysis will be performed according to different degrees of patient’s endometriosis. Endometriosis will be divided into mild degree (grades I and II) and severe degree (grades III and IV) due to the concern of false negatives and false positives.

Step 3: Multivariable analysis and sensitivity analysis

If the baseline data are not balanced between the two groups after randomization, we will adjust the unbalanced factors and perform a multivariable analysis. We will perform sensitivity analysis for loss of follow-up, missing data, which may bias experimental results.

#### Temporary analysis and data monitoring

Each quarter, the researchers at all centers will collect the data. Each center will collect its own data, and the researchers will communicate about whether there were any missing data or loss of patients to find the problems in a timely manner.

## Abbreviations

2PN: Two pronuclei
AMH: Anti-Müllerian hormone
ANOVA: Analysis of variance
CI: Confidence interval
COH: Controlled ovarian hyperstimulation
DMEC: Data monitoring and ethics committee
E2: Estradiol
FSH: Follicle-stimulating hormone
Gn: Gonadotrophin
GnRH: Gonadotrophin releasing hormone
HCG: Human chorionic gonadotrophin
ICSI: Intracytoplasmic sperm injection
IQR: Interquartile range
ISRCTN: International standardized randomized controlled trial number
IVF: In vitro fertilization
LH: Luteinizing hormone
MID: Minimally important difference
MII: Metaphase II
OHSS: Severe ovarian hyperstimulation
OR: Odds ratio
P: Progesterone
PGD: Preimplantation genetic diagnosis
PGS: Preimplantation genetic screening
PRL: Prolactin
RCT: Randomized controlled trial
REC: Regional ethics committee
SD: Standard deviation
ZZU: Zhengzhou University
